# Shear Stress and Endothelial Mechanotransduction in Trauma Patients with Hemorrhagic Shock: Hidden Coagulopathy Pathways and Novel Therapeutic Strategies

**DOI:** 10.3390/ijms242417522

**Published:** 2023-12-15

**Authors:** Athanasios Chalkias

**Affiliations:** 1 Institute for Translational Medicine and Therapeutics, University of Pennsylvania Perelman School of Medicine, Philadelphia, PA 19104-5158, USA; thanoschalkias@yahoo.gr; 2 Outcomes Research Consortium, Cleveland, OH 44195, USA

**Keywords:** blood flow, microcirculation, endothelium, endothelial cell, mechanotransduction, mechanosensing, coagulation, translational research, critical care medicine, centhaquine

## Abstract

Massive trauma remains a leading cause of death and a global public health burden. Post-traumatic coagulopathy may be present even before the onset of resuscitation, and correlates with severity of trauma. Several mechanisms have been proposed to explain the development of abnormal coagulation processes, but the heterogeneity in injuries and patient profiles makes it difficult to define a dominant mechanism. Regardless of the pattern of death, a significant role in the pathophysiology and pathogenesis of coagulopathy may be attributed to the exposure of endothelial cells to abnormal physical forces and mechanical stimuli in their local environment. In these conditions, the cellular responses are translated into biochemical signals that induce/aggravate oxidative stress, inflammation, and coagulopathy. Microvascular shear stress-induced alterations could be treated or prevented by the development and use of innovative pharmacologic strategies that effectively target shear-mediated endothelial dysfunction, including shear-responsive drug delivery systems and novel antioxidants, and by targeting the venous side of the circulation to exploit the beneficial antithrombogenic profile of venous endothelial cells.

## 1. Introduction

Massive trauma and severe hemorrhage are leading causes of hospitalization and disability in the world, contributing to more than 2.5 million deaths each year [1,2]. Research on models of mortality have shown that there are two patterns of death. Early deaths occur within the first hours or days after injury, due to severe cardiovascular collapse, including cardiogenic, hypovolemic and/or distributive shock. Late deaths usually occur during hospitalization, and are characterized by a persistent (severe) inflammation, immunosuppression and catabolism (PIIC) syndrome plus multiple episodes of sepsis [3,4].

Of note, one-third of the patients are coagulopathic upon arrival at the Emergency Department [1]. Trauma-induced coagulopathy (TIC) occurs early after injury and is biochemically evident prior to, and independent of, the development of significant acidosis, hypothermia, or hemodilution [5,6]. In general, the risk of coagulopathy increases with the severity of trauma [7].

## 2. Trauma-Induced Coagulopathy

After traumatic injury, coagulation, anti-coagulation, and fibrinolysis are disproportionally affected in a dynamic manner, leading to impaired hemostasis. Of note is that trauma is a heterogeneous event, and it is difficult to define a dominant mechanism of TIC [6,8,9]. The latter has been considered to be primarily due to blood loss from injury, hemodilution from aggressive resuscitation, and the development of hypothermia and acidosis [10,11].

Immediately after trauma, tissue injury exposes tissue factor to the circulation and initiates thrombin generation and clot formation. Platelets are activated through a network of regulated interconnecting cellular signals, including collagen in the sub-endothelial matrix binding to glycoprotein VI, von Willebrand Factor (VWF), and glycoprotein Ib [12]. The activation of platelets amplifies thrombin generation and the clotting process, causing the consumption of the coagulation factors, especially of fibrinogen and factor V [13]. Strikingly, during the acute post-injury phase, platelets develop a state of impaired ex vivo agonist responsiveness, independent of platelet count, associated with systemic coagulopathy and mortality risk. In addition, fibrinolysis is activated from the release of tissue-plasminogen activator (t-PA), which converts plasminogen to plasmin, into the circulation. Activated protein C plays a central role in enhancing anticoagulation; based on this hypothesis, thrombin is generated and binds thrombomodulin to form activated protein C following severe trauma injury and hypoperfusion. Activated protein C exerts its anticoagulant role by inhibiting factor Va and VIIIa and its hyperfibrinolytic role, by inhibiting t-PA inhibitor. Furthermore, tissue injury induces sympathoadrenal responses and catecholamine release, which damages the endothelial glycocalyx and converts the endothelial function from antithrombotic to prothrombotic for local hemostasis.

During the resuscitation phase, metabolic acidosis and hypothermia may develop, together with hemodilution from resuscitation fluids used to improve hemodynamics. These factors may further impair and amplify the already existing coagulopathy from the trauma injury. Acidotic trauma patients show impaired clotting enzyme activities, prolonged prothrombin and activated partial thromboplastin time, decreased coagulation factor levels, impaired thrombin generation, and depleted fibrinogen levels and platelet counts [14,15,16]. Also, hypothermia inhibits the initiation phase of thrombin generation, involving the formation of factor VII/tissue factor complex, and decreases fibrinogen synthesis via different mechanisms [17,18].

During the late post-traumatic phase, the systemic levels of cytokines and hormones increase, leading to endothelial cell activation and a slow transition of the endothelial cell phenotype from antithrombotic to prothrombotic. In patients surviving the initial insult, platelets become hyper-responsive, associated with increased risk of thrombotic events. Beyond coagulation, platelets constitute part of a sterile inflammatory response to injury, both directly through release of immunomodulatory molecules, and indirectly through modifying behavior of innate leukocytes [19,20,21]. Hypercoagulability ensues, owing to prothrombotic changes and fibrinolysis shutdown and/or activation of a persistent inflammatory state, promoting microvascular occlusion, tissue hypoperfusion, and organ failure [6,20]. Moreover, the production of extracellular vesicles, from multiple cellular sources, which are strongly prothrombotic, may result in coagulation factor depletion after injury [6]. In addition, the fibrinogen levels increase several folds, due to acute phase responses. The coagulation process at this phase becomes the prothrombotic state, predisposing patients to venous thromboembolism.

As stated earlier, coagulopathy occurs when procoagulants are consumed or diluted, and when one or more of the control mechanisms are disrupted [6]. However, shear-induced changes in cell-mediated regulation of hemostasis can also lead to or aggravate coagulopathy, as several events (initiation, amplification, and propagation) of thrombin generation are regulated by cell surfaces. Although early hypocoagulable coagulopathy differs mechanistically from iatrogenic coagulopathy [22,23], abnormal shear stress can affect both of these hypocoagulable phenotypes. In addition, shear stress induces membrane currents and increases the permeability of the cell membrane to extracellular calcium, thereby increasing its cytosolic concentration [24,25,26]. Therefore, abnormal shear stress may induce transients in calcium concentration and lead to hypocoagulable coagulopathy.

Hyperfibrino(geno)lysis, a combination of fibrinolysis and fibrinogenolysis, is caused by acute release of t-PA [27,28,29]. Tissue-plasminogen activator is released from the endothelium, due to decreased microcirculatory flow and via adrenaline, vasopressin, and thrombin signaling [6]. Of note, both microcirculatory flow impairment (low shear stress) and adrenaline- and vasopressin-induced vasoconstriction (high shear stress) may lead to shear-mediated aggravation of hypocoagulable coagulopathy, through increased inflammation and oxidative stress [30,31,32]. In contrast to other causes or phenotypes of coagulopathy, shear-induced coagulopathy may affect all organs.

## 3. Hemodynamic Perturbations in Massive Trauma

### 3.1. Macro- and Microcirculation

Hemorrhage decreases the stressed volume, and therefore venous return and left-ventricular end-diastolic volume, leading to arterial hypotension [33]. Initially, blood loss is largely compensated for by conversion of part of the unstressed into stressed volume, due to an increase in sympathetic discharge. Specifically, baroreceptor unloading leads to reflex tachycardia and increases peripheral vascular resistance, which maintain an effective circulation and tissue perfusion [34]. Clinically noticeable hypovolemia begins when the stressed volume starts to decrease and the unstressed volume has decreased to a physiologically possible maximum.

Patients with blood loss up to 15% of their total blood volume develop tachycardia and maintain mean arterial pressure. However, when blood loss exceeds 30% of total blood volume, a depressor phase becomes apparent, manifested as bradycardia and reduction in peripheral vascular resistance [35,36,37]. These patients have exhausted their physiological reserves, are unable to compensate for severe hypovolemia, and have severe circulatory failure [38,39].

The microcirculation, i.e., the most important physiological compartment of the cardiovascular system, where oxygen and nutrients are delivered to parenchymal cells, is also affected. The hemodynamic alterations described above significantly impede microcirculatory flow, leading to immediate compensatory readjustments in microvessels (vasoconstriction—vasodilation), which favor the maintenance of tissue perfusion. In bleeding patients, the relative hematocrit in small vessels decays as the vessel diameter decreases (Fåhræus effect). This decreases oxygen delivery, and the oxygen extraction rate increases until eventually the amount of oxygen that can be extracted plateaus [40]. From this point, any further decrease in oxygen delivery will cause oxygen consumption to decline, such that the value of oxygen consumption is now flow-dependent. In later stages of hemorrhage, severe hypovolemia leads to microcirculatory dysfunction, with loss of autoregulation. In addition to the aforementioned, it is particularly noteworthy that trauma-induced hemodynamic perturbations significantly affect the mechanical forces exerted on blood vessels, which may also induce shear-mediated abnormal microcirculatory responses.

### 3.2. Sympathoadrenal Activation

Increasing sympathoadrenal activation enhances perivascular sympathetic nerve exocytosis of catecholamines and the release of active tPA into the vessel walls and the microvasculature [41]. The catecholaminergic surge causes endothelial damage/dysfunction, glycocalyx shedding, breakdown of tight junctions with capillary leakage, and hyperfibrinolysis [41,42,43]. A study with more than 3000 patients with different types of acute critical illness, including different types of shock, reported strong and independent associations between high injury severity, high plasma adrenaline level, profound hypocoagulability, and high circulating syndecan-1 levels [43]. Together with capillary leak, perivascular edema, and vasoconstriction, these responses provoke tissue hypoperfusion and hypovolemia, and are predictive of organ failure and death [41,43,44,45]. In another study in rats, chemical sympathectomy diminished inflammation, glycocalyx shedding, and coagulopathy [46]. Therefore, a complex relationship seems to exist between sympathoadrenal activation, inflammation, and coagulopathy [45,46,47]. While both norepinephrine and epinephrine degrade the glycocalyx and damage the endothelium in a dose-dependent fashion, norepinephrine induces a fibrinolytic state with elevated tPA levels, compared to epinephrine, while the latter appears to produce more severe endothelial instability [44].

### 3.3. Endotheliopathy of Trauma

Endotheliopathy of trauma (EoT) is a systemic response mediated by hypoperfusion, and may be evident within five to eight minutes of injury [48]. It is characterized by loss of barrier function, leukocyte adhesion, micro- and macrothrombosis, and organ dysfunction [49,50]. Sheddase-induced degradation and loss of glycocalyx activates the endothelium and disrupts tight and adherens junctions, which worsens shock physiology and further propagates coagulopathy. In turn, TIC aggravates EoT, forming a vicious pathophysiological cycle [51]. Clinically, this is manifested as a pro-inflammatory state with vascular leak and tissue edema, which contributes to organ failure, late morbidity, and deaths [52].

Recent advances in hemostatic resuscitation increased our knowledge on how to mitigate EoT and repair the dysfunctional endothelium. Administration of crystalloids aggravates glycocalyx damage, increases plasma syndecan-1 levels, and worsens permeability [53], while albumin may only partially restore endothelial glycocalyx thickness [54]. Polyethylene glycol-20K (PEG) can establish an osmotic gradient from swollen cells to capillary lumens, facilitating capillary fluid shift and volume expansion, and thus it may be a better option than albumin for prolonged care of hemorrhagic shock [55]. Also, fresh frozen plasma may completely restore endothelial glycocalyx thickness, but may not be helpful in increasing shed syndecan-1 levels [54,56,57]. In addition, administration of fibrinogen may have a protective effect on the endothelial barrier [58,59,60,61].

## 4. Mechanical Forces Exerted on the Vasculature

### 4.1. Endothelial Surface Mechanosensing

The endothelium that lines the vascular wall is an extensive and dynamic organ that serves as an interface where chemical and mechanical stimuli in blood interact directly with a cellular layer [61]. In adults, the entire endothelium has a surface area of 3000 m^2^, which is equivalent to seven professional basketball courts [62,63]. Vascular endothelial cells are situated between the flowing blood and the vessel wall, and are directly exposed to blood flow. In humans, normal physiological flow is ~1–5 Pa (one Pa is equal to one N m^−2^ and 10 dynes cm^−2^), and is highly pulsatile in arteries, of a similar magnitude but less pulsatile in capillaries, and about 10-fold less, with minimal pulsatility, in veins [30]. These forces are very low, compared to typical traction stresses (~5 kPa) and artery wall strains (~100 kPa) [64,65], which underscores the sensitive nature of these endothelial sensing mechanisms [66]. Impressively, the mechano-remodeling of endothelial cells is responsible for their elongation in the direction of the undisturbed flow [67].

Blood ejected from the left ventricle exerts two main forces on the endothelium lining the cardiovascular system: the frictional force from blood flow (i.e., fluid shear stress) that is parallel to the vessel wall, and the force from blood pressure that distends the vessel wall. Other authors suggest that there are three major types of mechanical stress: the cardiac cycle-generated hemodynamic forces that are perpendicular to the endothelial surface, the tangential forces (e.g., shear stress), and the circumferential stretch [68,69]. The vessels themselves also contribute to the application of mechanical forces on their inner wall, due to their elasticity, which helps them recover their size and shape (recoil) after being stretched by an aliquot of blood, pushing the aliquot downstream [70,71,72]. These flow-induced mechanical forces guide the physiology of the human body and are particularly crucial for preserving the function of the cardiovascular system [61,73].

**Figure 1:**
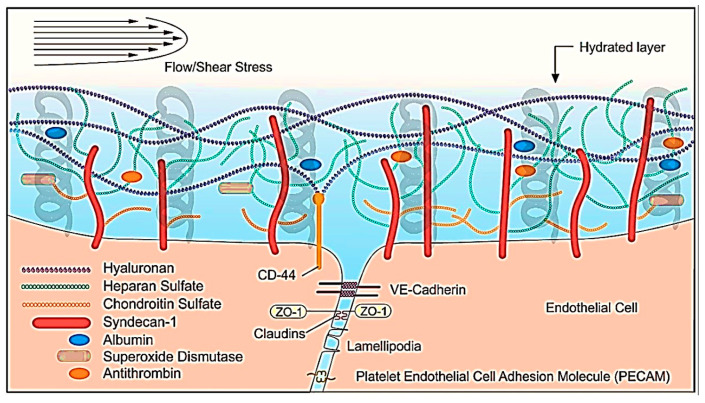
Exposure of healthy endothelium and glycocalyx layer to physiological blood flow patterns. This dynamic organization acts as a “suspension” (embedded in Figure for didactic purposes), enabling mechanotransduction, i.e., the conversion of mechanical stimuli generated by blood flow to biochemical signals that elicit specific cellular responses. Normal microcirculatory flow enhances the expression of multiple anti-inflammatory, antithrombotic, and antioxidative mediators to stabilize the vessel wall, and thus maintain an antioxidant, anti-inflammatory, and antithrombotic state. Modified from Reference [79] under the terms and conditions of the Creative Commons Attribution (CC BY) license.

A hydrated layer comprising the glycocalyx and other molecules, e.g., angiotensin-converting enzyme, located on the inner surface of the vascular endothelium, helps endothelial cells adapt to normal blood flow and flow changes, maximizing the overall functional endothelial performance [73,74,75,76,77,78,79]. We can imagine this layer as a ‘suspension system’ receiving the flow-induced mechanical stimuli (Figure 1). In turn, endothelial glycocalyx is also a dynamic multilayered structure, adapting to surrounding environmental demands by functioning as a mechanosensor and transducer [68]. Glycocalyx functions as a “Wind in the Trees” model, where the wind (or force) is sensed by the outer branches and transmitted through the cell membrane, ultimately resulting in intracellular signaling. For example, results from animal studies show that the application of a steady amount of shear stress to the vessel wall increases nitric oxide production [69].

The dynamic organization of the hydrated layer is dependent on adequate shear stress from flowing blood, and enables mechanotransduction and modulation of capillary (endothelial) permeability [61,66,74,75,76,77,78,79]. Normal shear stress levels from laminar flow have a powerful regulative effect and contribute significantly to maintaining local homeostasis, vessel physiology, and tissue perfusion [54]. In healthy individuals, however, shear stress varies, according to the vascular bed (Table 1) [61,80,81,82,83,84].

Endothelial cells sense and respond to normal flow-mediated mechanical stimuli by converting them to biochemical signals, enhancing the expression of multiple anti-inflammatory, antithrombotic, and antioxidative mediators, which stabilize the vessel wall and control apoptosis [85,86,87,88]. The stretch and shear stress resulting from circulatory pressure and flow, respectively, modulate endothelial cell functions by activating mechanosensors that regulate gene and protein expression and cell behavior via specialized mechanisms and signaling pathways (mechanotransduction) [66,85,89,90,91,92,93]. Specifically, endothelial cells possess sophisticated mechanosensing abilities to detect distinct features of flow profiles, including magnitude, direction, amplitude, and frequency of flow [94]. They are known to have approximately 15 mechanosensors that modify 10–20 signaling pathways, which, in turn, regulate the expression of 1000–2000 genes. Interestingly, microarray studies with cultured cells and in vivo studies have shown that the majority (90%) of endothelial cell genes are regulated by the steady component of blood flow, and only 10% are regulated by pulsatile conditions [95].

### 4.2. Endothelial Nuclear Mechanosensing

As the largest and stiffest organelle, the nucleus plays an important role in mechanosensing and mechanotransduction. The relevance for the endothelium as a paradigm for studying nuclear mechanosensing was indicated by experiments in which isolated nuclei were exposed to shear stress. This changed the nuclear lamina (lamin A/C, encoded by LMNA), leading to stiffer nuclei [96]. Because of the thin, flat morphology of endothelial cells, the nucleus is elevated above the rest of the cell body, and thus is primarily exposed to shear stress, creating hydrodynamic drag [97]. Tkachenko et al. suggested that this drag on the nucleus changes the cytoskeleton to confer tension on the mechanosensory junctional complex and trigger flow-responsive signaling, dictating polarity and positioning both the nucleus and the microtubule-organizing center [97]. Shear stress can increase stiffness, decrease height, and align or remodel nuclei parallel to the direction of flow; in this capacity, nuclei align or remodel themselves to limit the exposure to mechanical force, in order to protect genomic integrity [96,98,99]. Individual components of the nuclear membrane determine the appropriate response of endothelial cells to mechanical force, such as nesprin 1 (SYNE1), 2 (SYNE2), and 3 (SYNE3) [96].

Recent work indicated communication from the LINC complex to junctions via a microtubule-SUN1 connection to modulate endothelial barrier function [100]. Also, the junctional molecule angiomotin-like protein 2 (AmotL2) binds VE-cadherin and p120 catenin in a complex to link SUN2 via the actin cytoskeleton, a connection that is required for shear stress-mediated endothelial cell alignment [101]. Overall, these emerging studies suggest a continuous link between cell–cell junctions and the nuclear membrane that informs nuclear dynamics and endothelial (and epithelial) responses to mechanical force.

Cooperation between mechanosensors has been documented by numerous laboratories, showing that junctional mechanosensing can lead to the activation of integrins and focal adhesions. Some authors studied specific mechanosensors, such as Piezo1, and the cooperation between junctions and GPCRs [102,103]. Precisely how these mechanosensors cooperate with the nucleus to inform wider transcriptional adaptation to force is less-well understood. The emerging evidence discussed above indicates that junctions cooperate with the nucleus in sensing mechanical forces via the cytoskeletal network [100,101]. Further research is therefore needed to understand the importance of primary mechanosensors in bidirectional communication with the nucleus, via other signaling networks.

### 4.3. Effects of Shear Stress on Endothelial Cell Junctions

Regulation of microcirculatory blood volume and velocity of red blood cells (RBCs) requires tight control of blood flow by resistance arteries. These vessels respond to changes in shear stress and transmural pressure by regulating their vascular tone and luminal diameter, maintaining a constant capillary flow [104,105]. In turn, regulation of vascular tone and luminal diameter requires a healthy endothelium detection and communication between the vascular endothelium and smooth muscle cells [104]. Endothelial cells form multiple junctional complexes with their neighbors, including adherens junctions, tight junctions, and gap junctions, as well as adhesion mediated by binding of the platelet endothelial cell adhesion molecule-1 (PECAM-1) [106].

Shear stress is sensed through a variety of mechanisms, the most well-known being the VE-cadherin (or CDH5), PECAM-1, VEGFR-2 (or KDR), and VEGFR-3 (or FLT4) mechanosensory complex within endothelial cell–cell junctions [93,96]. This complex is activated when shear stress induces PECAM-1-dependent activation of the nonreceptor tyrosine kinase Src, which then facilitates ligand-independent activation of VEGFR-2. VEGFR-2 phosphorylation triggers downstream activation of the p85 subunit of the phosphoinositide 3-kinase family, AKT kinases, and integrins [96]. Also, VEGFR-3 acts as a fluid shear-stress sensor that regulates inward vascular remodeling, which is crucial in maintaining vascular homeostasis and regulating shear stress [107].

The transmembrane receptor plexin D1 plays an important role in directly sensing fluid shear stress, as conformational change of this receptor is required for downstream mechanotransduction [108]. Specifically, binding of plexin D1 to neuropilin 1 and VEGFR-2 occurs under shear stress, and triggers downstream flow-responsive signaling, highlighting plexin D1 as an upstream mechanosensor in the junctional mechanosensory complex [108]. Recently, another junctional shear stress-sensing molecule, the AmotL2, was identified, which binds VE-cadherin and p120 catenin (CTNND1) in endothelial cells [101]. Neuropilin 1 was also shown to bind VE-cadherin in a flow-dependent manner, promote flow-induced association of p120 catenin and VE-cadherin, and regulate downstream anti-inflammatory signaling [109].

Two prominent endothelial sensors of shear stress are the Piezo-1 channel (named from the Greek “πίεση” (piesi) meaning pressure) and the PECAM-1 cell adhesion molecule. Recent evidence suggests a pool of Piezo-1 at cell junctions, which interacts with PECAM-1 and adhesion molecules in tailoring the junctional structure to mechanical requirements [102]. However, altered shear stress induces reorganization of cytoskeleton and junctional structures, leading to shape change and cell reorientation. Changes to adherens junctions may occur when shear stress changes chronically, possibly reflecting adaptations that enhance adhesion between cells that are exposed to increased mechanical loads. Indeed, shear stress may cause partial disassembly of the adherens junction complex, followed by a reassembly that reflects shear-induced adaptive reorganization of the actin cytoskeleton [106]. However, the evolving hemorrhage and shock maintain abnormal shear rates, leading to endothelial injury [106]. Adherens junctions in arterial bends and branch sites are particularly vulnerable to hemodynamic perturbations, and their destruction is a characteristic feature of endotheliopathy and vascular integrity. Therefore, targeting the venous side of the circulation using novel venoconstrictors may prevent shear-induced adverse events, and improve outcomes.

### 4.4. Effect of Arterial Stiffening on Endothelial Mechanosensing

The native residents, endothelial cells and vascular smooth muscle cells, are not only affected by various chemical factors, including inflammatory mediators and chemokines, but also by a range of physical stimuli, such as shear stress and extracellular matrix (ECM) stiffness, presented in the microenvironmental niche. For example, blood arteries undergo stiffening and lose their elasticity with age. Reports have shown that the ECM stiffening could influence the fate of endothelial cells by changing the cell adhesion, spreading, proliferation, cell-to-cell contact, migration, and even communication with smooth muscle cells [110]. Because the ECM is a highly dynamic structure that constantly undergoes remodeling, changes in the physical microenvironment of ECM play a significant role in endothelial cell viability [110].

### 4.5. Effects of Shear Stress on Blood Cells

The presence of RBCs has a significant effect on the wall shear stress, especially in the microcirculation. The effect becomes more dynamic under conditions that induce fluctuations in RBC morphology [111]. Of note, the high deformability of RBCs is an essential element of blood fluidity, and modulates RBC interactions with blood vessel walls and other blood cells [112]. Under physiological conditions, the maintenance of RBC deformability by normal shear forces facilitates microcirculatory perfusion and oxygen delivery [113]. However, the aggregation of RBCs and their structure are sensitive to shear changes, and extreme shear conditions can lead to irreversible RBC damage and endothelial hypoxia [114,115]. Oxygen transport to tissue is further aggravated by the concentration of fast-flowing RBCs in the center of the lumen, and of slower-flowing plasma along the wall of the vessel. In patients with trauma, RBC aggregation is further affected by shear-induced platelet aggregation [115]. These phenomena underline the role of RBCs, the presence of which influences the magnitude of endothelial-wall shear stress. However, the vast majority of studies of endothelial responses to flow do not account for the impact of RBCs.

Leukocytes are able to sense normal shear stress, as well as remarkably low shear stresses. Normal shear stress serves to reduce activation, pseudopod projection, and expression of adhesion molecules of circulating leukocytes. In trauma, the injured endothelium expresses a number of membrane adhesion receptors to capture circulating leukocytes, the majority of which are in a nonactivated state without pseudopods. However, hemorrhage-induced low shear stress leads to pseudopod projection and the spreading of leukocytes on the endothelium [116]. After capture and adhesion to the endothelium, the leukocytes experience a sudden and substantial increase in fluid shear stress on their surface, which may be a key signal to the cell to initiate migration. Activated circulating leukocytes can also cause a significant flow reduction in capillaries, to the point of complete stasis and microvascular entrapment [116], further aggravating the shear-induced endotheliopathy. Notably, it is equally possible that leukocytes could initiate migration in abnormally high shear rates, although it remains unknown if leukocytes project or retract pseudopods in response to pressures higher that 1000 dyn cm^−2^.

A shear stress of 170 N m^−2^, acting for as short a time as 7 milliseconds, makes available procoagulant phospholipids and activates platelets [117]. Platelet adhesion to the subendothelium at high shear stresses requires transient rolling, mediated by weak, short-lived GPIb-von Willibrand factor (VWF) bonds, followed by firm adhesion by strong, long-lived bonds mediated by activated integrins [118]. However, abnormally high shear causes shedding of GPIbα and GPVI, and decreases margination and the number of platelets adhered on VWF and collagen in a shear- and time-depended manner [119,120]. Also, platelet margination is caused by platelet interactions with RBCs in flowing blood, which are decreased in patients with hemorrhagic shock [118]. Thus, it is hard for platelets to form firm adhesion to the endothelial wall, a factor which compromises blood clotting in these individuals.

## 5. Low Endothelial Shear Stress in Patients with Hemorrhagic Shock

Tissue injury and hemodynamic alterations significantly change the main forces exerted on the vasculature. In turn, vascular endothelium is highly sensitive to variations in shear stress, which induce structural wall remodeling [121,122]. Therefore, mechanotransduction can contribute to the development and aggravation of endothelial dysfunction when either the forces themselves, or the mechanisms by which forces are sensed, are pathological [61,123].

In trauma patients with significant blood loss, hypotension, impaired microcirculatory autoregulation, tissue injury, aggressive resuscitation, transfusion, and changes in viscosity are associated with abnormal shear stress on endothelial cells. When the latter are exposed to low, abnormal shear forces, local homeostasis is lost and the endothelium begins to remodel, displaying a hypercoagulant/prothrombotic and pro-oxidant state. Increases in glycocalyx shedding and vascular permeability correlate with larger reductions in blood flow, as demonstrated in the postcapillary venules of skeletal muscle and mesentery of rats [43,54,124]. Furthermore, loss of the endothelial barrier leads to the exposure of procoagulant and platelet-activating proteins, the shedding of glycocalyx components, hypocoagulability, and the release of fibrinolytic factors promoting fibrin(ogen)olysis [8,66,121,125].

**Figure 2:**
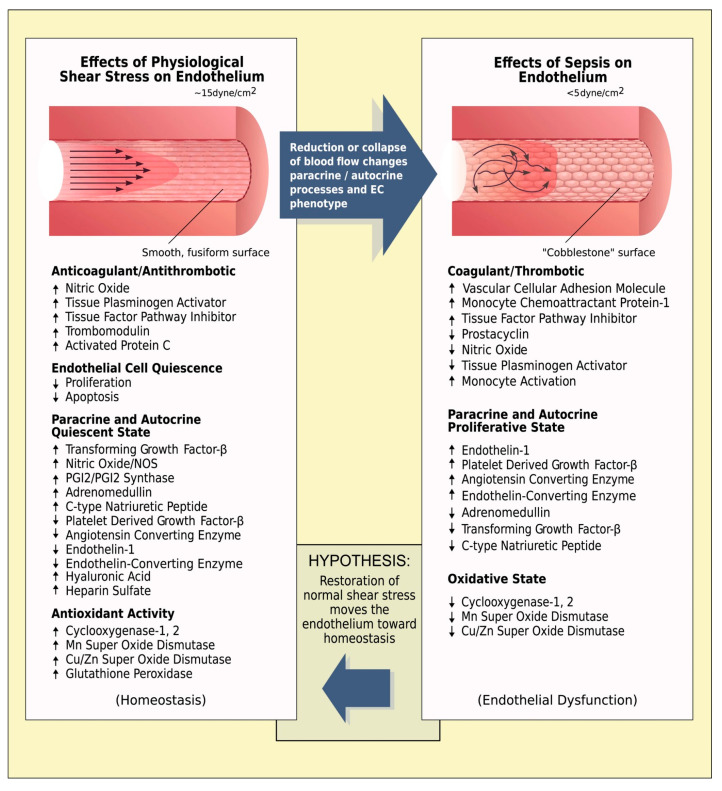
Effects of normal and reduced shear stress on the vessel wall. Left panel: normal endothelium receiving physiological (~15 dynes cm^−2^) shear stress from laminar blood flow patterns. The endothelium exhibits a quiescent fusiform smooth morphology, with increased production of substances that are anticoagulant/antithrombotic and antioxidant. The endocrine status is summarized also, supporting the quiescent status. Right panel: dysfunctional endothelium resulting from low shear stress (<5 dynes cm^−2^). The endothelium displays a hypercoagulant/prothrombotic and pro-oxidant state. Autocrine and paracrine changes listed contribute to this dysfunctional vascular organ. In vitro and in vivo studies suggest that restoration of laminar blood flow and adequate shear stress would move the endothelium towards homeostasis. Reproduced from Reference [79] under the terms and conditions of the Creative Commons Attribution (CC BY) license.

Research on sepsis has also shown that inadequate shear stress impairs the shear-mediated mechanotransduction, and initiates pathological changes [79]. Specifically, normal endothelium receiving physiological (~15 dynes cm^−2^) shear stress from laminar blood flow patterns exhibits a quiescent fusiform smooth morphology, with increased production of substances that are anticoagulant/antithrombotic and antioxidant [126]. However, dysfunctional endothelium resulting from low shear stress (<5 dynes cm^−2^) displays a cobblestone appearance and a hypercoagulant/prothrombotic and pro-oxidant state (Figure 2) [79,127,128].

Similar phenomena occur in patients with trauma; they are evident from the time of injury, and may persist for as long as microcirculatory impairment remains significant [129,130]. Of note, both sepsis and hemorrhage are characterized by an initial hyperdynamic phase including vasoconstrictive responses, followed by a hypodynamic one characterized by profound vasodilation. In patients with physiological reserves, however, alternations of vasoconstriction and vasodilation may also be observed, even within the same organ. Therefore, the effects of shear changes are likely to be similar, especially in the initial stages of both conditions.

In addition, hemorrhage can develop lethal inflammatory responses or systemic inflammatory response syndrome (SIRS), similar to that of sepsis [131]. Although hemorrhage activates similar responses though an intrinsic response to tissue damage, and sepsis represents an extrinsic activation in response to infection or bacterial endotoxins, it is the balance of pro- and anti-inflammatory mediators derived from the innate immune system that defines the progression and severity of both conditions. Consequently, in both phenomena, overwhelming inflammatory responses can become more dangerous than the original pathogenic insult [131]. Specifically, an overproduction of endogenous proinflammatory mediators, including cytokines, platelet-activating factor, oxygen radicals, and nitric oxide, synergistically interact to mediate hypotension, multiple organ failure, and death [131]. This is more notable in intensive care unit trauma patients with postinjury sepsis, in whom the mechanistic changes at the cellular and molecular level remain poorly understood [132].

## 6. High Endothelial Shear Stress in Patients with Hemorrhagic Shock

Increases in shear stress to abnormal levels also have adverse effects on endothelial function. Recent data from studies with small and large vessels from mice and healthy human donors suggest that (various types of) lesions result in high shear rates and elongational flows at the edge of the wound where hemostasis occurs [133]. Notably, such shear levels not only far exceed those observed in healthy vessels under homeostatic conditions, but can also damage adjacent healthy vessels [133,134]. High shear stress or dysregulated flow also affects glycocalyx thickness and its composition [135,136].

In addition, post-traumatic vasoconstriction, due to physiological compensatory responses, severe pain, vasopressor support, and/or aggressive resuscitation efforts may also generate high shear rates, leading to morphological changes in platelets [135]. These changes occur even if high shear forces are applied for only a few milliseconds to platelets, and affect receptor–ligand binding interactions and platelet margination [117,120]. As a result, platelets cannot form firm adhesion to the wall and participate in thrombus formation. In some individuals, high shear may lead to platelet activation, and increase their adhesion with fibrinogen [117,119].

**Figure 3:**
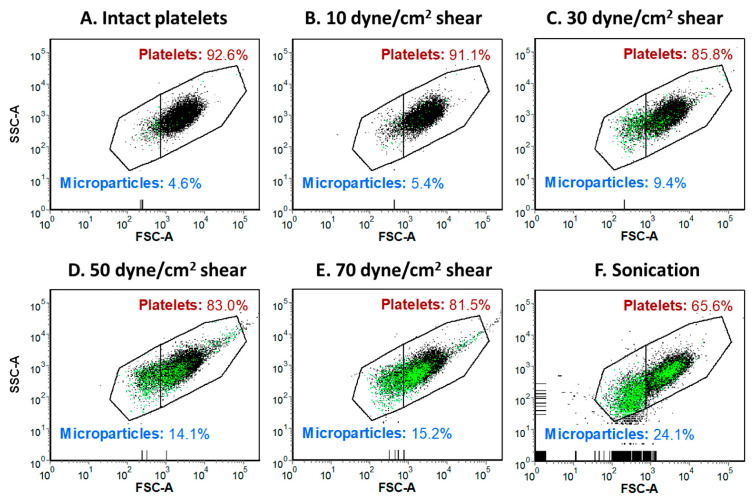
Platelet exposure to shear stress and sonication induces increased generation of platelet-derived microparticles. Illustrative dot diagrams of αIIb+ platelets and αIIb+ microparticles, gated based on their forward- and side-scatter characteristics (**A**–**F**): (**A**)—intact platelets, (**B**–**E**)—sheared platelets, (**F**)—sonicated platelets. Black dots—CD41+ platelets or microparticles. Green dots—CD41+/annexin V+ double positive microparticles. FSC-A—forward-scatter amplitude. SSC-A—side-scatter amplitude. Reproduced from Reference [137] under the terms and conditions of the Creative Commons Attribution (CC BY) license.

**Figure 4:**
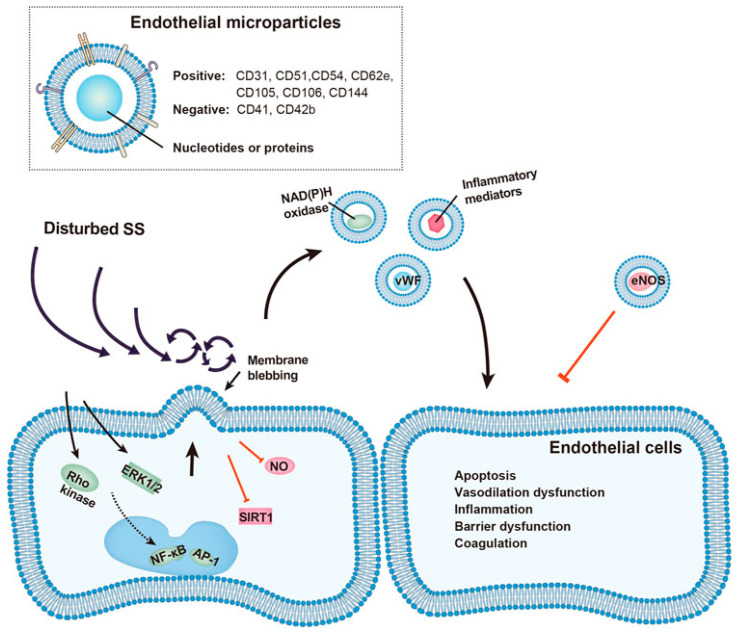
Schematic representation of vascular injury by endothelial microparticles under disturbed shear stress. Endothelial microparticle release is increased under disturbed shear stress through activation of Rho kinase, ERK1/2, NF-κb, and AP-1 pathways, or by inhibition of STIR1 and nitric oxide. Released endothelial microparticles, identified by presence of endothelial and absence of platelet surface markers, carry functional proteins including von Willebrand factor, NAD(P)H oxidase, inflammatory mediators, and endothelial nitric oxide synthase. These endothelial microparticles affect nearby endothelial cells and promote vascular apoptosis, vasodilation dysfunction, inflammatory response, barrier dysfunction, and local hypercoagulation. SS, shear stress; NO, nitric oxide. Reproduced from Reference [138] under the terms and conditions of the Creative Commons Attribution (CC BY) license.

Exposure to supraphysiologic shear stress also leads to platelet membrane thinning and the generation of sheared platelet-derived microparticles that promote thrombin generation and inhibit collagen- and adenosine diphosphate-induced platelet aggregation (Figure 3) [137]. Shear-mediated microvesiculation is also associated with the remodeling of platelet receptors, with microparticles expressing significantly higher levels of adhesion receptors (αIIbβ3, GPIX, PECAM-1, P-selectin, and PSGL-1) and agonist receptors (P2Y12 and PAR1) [137]. Furthermore, high shear can remarkably increase the release of endothelial microparticles that impair vascular senescence, inflammatory response, barrier function, and local coagulation, enhancing the injurious impact of disturbed shear stress on the vasculature (Figure 4) [138]. Similar to platelet-derived microparticles, endothelial microparticles can affect adjacent endothelial cells, and aggravate local injury [139,140,141,142]. The aforementioned functional changes can be manifested clinically as bleeding and thrombotic adverse events.

Although hypothermia reduces cellular oxygen demands and protects vital organs during periods of cellular ischemia [143], it may aggravate coagulopathy, reduce both plasma volume and circulating blood volume, and worsen tissue perfusion [144]. Rewarming increases the metabolic rate and improves tissue perfusion by enhancing endothelial-dependent dilation (flow-mediated dilation) via greater bioavailability of nitric oxide [145,146]. However, the exact associated mechanisms are unclear. Rewarming is also associated with dysfunction of the efferent sympathetic nervous system, circulatory failure, and other complications [144,147]. Although adequate shear stress is an important hemodynamic stimulus for vascular adaptation, shear stress induced by acute exposure to (lower or) higher temperatures is not obligatory to protect against endothelial ischemia-reperfusion injury [148]. Of note, different shear rates may not modify flow-mediated dilation for a given heating stimulus [145,149].

Increases in core temperature via whole-body heating may significantly increase heart rate and—possibly—cardiac output in hypovolemic patients, causing a larger cumulative increase in shear stress in the arterial bed [145]. All these may enhance the (already abnormal) shear forces induced by local vasoconstriction and/or administration of vasopressors, aggravating endothelial dysfunction. Furthermore, shear-induced changes in blood viscosity and increases in oxidative stress may reduce bioavailability of nitric oxide, enhancing vasoconstriction and further impairing flow-mediated dilation and endothelial function [145,150]. As hemodynamic forces in the arterial circulation modulate endothelial function in conduit and resistance vascular beds [148], maintaining and/or improving endothelial homeostasis (in part by avoiding severe perturbations in blood flow) in these vessels is necessary for optimizing the effects of temperature on shear levels.

## 7. Novel Pharmacologic Approaches Targeting Shear-Mediated Endothelial Dysfunction

Significant progress has been made in recent years toward understanding the sequence of molecular events associated with endothelial mechanotransduction at abnormal shear rates. However, the wide range of operative mechanisms that regulate endothelial cells’ response to shear stress make it imperative to develop innovative pharmacologic strategies that effectively target shear-mediated endothelial dysfunction.

### 7.1. Shear-Responsive Drug Delivery Systems

Shear-responsive drug delivery systems have been explored as a potential approach for targeted administration of medications to lower systemic drug levels. For example, mechanosensitive liposomes consist of an active compound encapsulated in shear-stress-sensitive liposomes that release their payload at enhanced forces [151]. Another approach is a shear-activated nano-therapeutic aggregate (SANT) that breaks up into multiple smaller nanoparticles in a high-shear-stress environment [152]. The use of shear-responsive drug delivery systems has the potential to improve the efficiency and specificity of drug delivery, e.g., localized vasodilators, antithrombotics, and vasopressors, as the release of the drug can be precisely controlled in response to changes in shear stress [152,153,154]. These approaches may be effective for targeting the arterial system, in which the shear stress is significantly higher compared to that in the veins. Moreover, considering that the peak shear stress occurs near the vessel wall, shear-responsive drug delivery systems could also target the arterial endothelium. However, these approaches cannot currently overcome the washout effect for some drugs; i.e., tPA is a fibrin-targeted molecule, and its activity depends on binding fibrin, so it will exert local effects, while for drugs not targeted by nature (e.g., nitroglycerin), local release will be followed by rapid washout downstream [154]. These systems have a variety of clinical challenges and limitations, have not yet been approved for use in patients, and are still under investigation.

### 7.2. Antioxidants

In the setting of hemorrhagic shock, glycocalyx shedding is not distributed homogenously among all vascular beds, but is most prominent in specific organs, e.g., in pulmonary and intestinal vasculature, due in part to the greatest level of generation of reactive oxygen species within their endothelium [155]. Indeed, increasing evidence suggests that reactive oxygen species have a crucial role in shear signaling, and that bolstering the antioxidant defense can facilitate endothelial protection [61].

The most-studied antioxidant agent is resveratrol (trans-3,4′,5-trihydroxystilbene), a natural polyphenol phytoalexin with multiple cardiovascular and metabolic effects. Several authors report the ability of resveratrol to reduce abnormal shear-induced oxidative stress in endothelial cells [156,157]. In a rodent model of hemorrhagic shock (mean arterial pressure 40 mm Hg for 90 min), resveratrol (30 mg kg^−1^) administered during resuscitation prevented the overproduction of superoxide radical/NADPH oxidase expression and restored the trauma hemorrhage-impaired endothelium-dependent relaxation via stimulation of hemeoxygenase-1 expression [158]. Resveratrol can also ameliorate oxidative stress from low shear stress by suppressing the extracellular signal-regulated kinase/endothelial nitric oxide synthase-Thr495 in endothelial cells [156]. In addition, resveratrol, tannic acid, and statins may increase KLF2 expression, driving an anti-inflammatory phenotype in the endothelium [61,159,160].

At low concentrations, nitric oxide acts as a paracrine-signaling molecule, mediating vasodilation, inhibition of platelet activation, monocyte and leukocyte adhesion, and smooth muscle cell proliferation [161]. Abnormal shear stress modulates the activity of endothelial nitric oxide synthase and redox-sensitive transcription factors, both in vitro and in vivo, and prolonged therapeutic intervention with antioxidants and L-arginine can normalize this disequilibrium. Indeed, several studies have shown that long-term oral administration of L-arginine may improve nitric oxide-dependent vasodilation, reduce endothelial leukocyte adhesion and platelet aggregation, and attenuate vascular smooth muscle cell proliferation [162]. Also, intra-arterial infusion of vitamin C may decrease endothelial dysfunction and attenuate the reduction in repeated flow-mediated dilation following ischemia-reperfusion injury [163]. Combining antioxidants (e.g., a cocktail containing vitamins C, E, and a-lipoic acid) could perhaps provide a higher level of protection from ischemia-reperfusion injury than vitamin C in isolation [163]. Another important and potentially useful therapeutic approach for shear-induced oxidative stress could be to target antioxidants towards the mitochondria, with lipophilic cations such as mitoquinone (MitoQ) or MitoE2 [164]. However, some studies reported that MitoQ may be pro-oxidant and pro-apoptotic, because its quinone group can participate in redox cycling and superoxide production [164]. Enzymatic systems or cell-permeable cationic peptides directed to the mitochondria or nuclear factor erythroid 2-related factor 2, a redox-sensitive cell-protective transcription factor, may prove alternative therapeutic approaches [165].

### 7.3. Vasopressor Therapy

The effects of tissue injury and hemorrhage-induced flow perturbations at the microcirculatory and endothelial/molecular level are complex. At the initial stages of hemorrhage, the human body responds to hypovolemia (low shear forces) and decreased oxygen delivery with tachycardia and peripheral vasoconstriction. The latter may lead to turbulent flow and increased shear rates in microvessels. At later stages, the predominant hemodynamic disorder is sympathoinhibitory vasodilation, which results in low shear forces. In these patients, the administration of exogenous vasopressors may cause severe vasoconstriction and increase the shear rate to supranormal levels, especially in those who require higher blood-pressure targets, e.g., in patients with traumatic brain injury. Therefore, the course of severe bleeding is usually characterized by alternating vasodilation and vasoconstriction, leading to abnormally low or high shear rates, respectively, and exacerbating local injury and trauma-induced coagulation pathology.

#### 7.3.1. Non-Hemodynamic Effects of Catecholamines

Translational research during the last decades has shown that catecholamine-mediated stimulation of vascular endothelial adrenoreceptors hastens blood coagulation, through the release of coagulation molecules stored in endothelial cells [166,167,168,169,170,171,172,173,174]. Catecholamines drive the coagulation–fibrinolysis balance toward hypercoagulability, in a mostly β2-mediated fashion. Accelerated thrombus generation via platelet activation is also mediated by α2-adrenoceptors [175,176]. In addition, catecholamine-induced shear stress may promote platelet–platelet and platelet–vessel-wall interaction via several mechanisms, including combined α2- and β2-adrenoreceptor stimulation [177,178,179].

The most-studied catecholamine to date is epinephrine. A dose-dependent stimulation of factor VIII clotting activity, VWF antigen, and t-PA has been observed within a 15-to-40 min infusion of epinephrine [175]. In addition, epinephrine infusion induces functionally active factor VIII from the spleen, as well as short-term recruitment and activation of platelets [180,181].

Norepinephrine, the currently recommended first-choice vasopressor in hypotensive trauma patients, predominantly enhances α-adrenoceptor stimulation and has a mild effect on β1-adrenoceptors [182]. The β2-adrenergic effects of norepinephrine are less defined, and may occur with high concentrations of this drug [183]. Therefore, norepinephrine may also stimulate several pro-coagulopathy pathways in patients with massive trauma. In general, catecholamines may exert both separated and combined α- and β-stimulation, promoting a generalized prothrombotic effect in the human body.

#### 7.3.2. Targeting the Venous Side of the Circulation

Catecholamines affect the entire vasculature, as a result of the ubiquitous presence of adrenergic receptors in arteries and veins, raising significant concerns regarding their non-hemodynamic effects, including those induced by abnormal shear stress. As mentioned previously, normal physiological flow is ~1–5 Pa and highly pulsatile in arteries, of a similar magnitude but less pulsatile in capillaries, and about 10-fold less, with minimal pulsatility, in veins [30]. In addition, the majority (90%) of endothelial genes seem to be regulated by the steady component of blood flow, and only 10% are regulated by pulsatile conditions [95]. Considering that venous endothelial cells are less thrombogenic than arterial endothelial cells [184], maintaining a steady, non-pulsatile flow in the venous system, whilst avoiding a direct effect on the arterial system, seems intriguing. Therefore, targeting the venous side of the circulation may prevent the activation of pro-coagulant pathways or cascades induced by abnormal arterial shear stress in trauma patients.

#### 7.3.3. Centhaquine

Centhaquine is a novel agent being developed for use in the treatment of hemorrhagic shock. It acts on α2B-adrenoreceptors, which are abundantly present in veins whilst scarcely present in arteries, contributing most to venoconstriction [185]. In addition, centhaquine activates central α2A-adrenoreceptors, reducing systemic vascular resistance and afterload by attenuating sympathetic outflow. Therefore, centhaquine increases venous return and cardiac output, while facilitating arteriolar flow. The net effect of centhaquine is an increase in mean arterial pressure and tissue perfusion. This is confirmed by several experimental studies and clinical phase I (NCT02408731), II (NCT04056065), and III (NCT04045327) trials, which showed the superior effectiveness of centhaquine, compared to commonly used resuscitative agents, in reducing mortality following hemorrhagic shock [185,186].

Centhaquine has shown no significant binding to β-adrenergic receptors, and thus its effect on the cardiac output-related mechanical forces is meager [185]. Notably, no other in-use vasoactive compounds have shown similar effects till now. Therefore, centhaquine may prevent an increase or decrease in arterial shear stress to non-physiological levels.

Interestingly, novel evidence sheds more light on centhaquine’s mechanism of action. Studies in rats investigating its effects on the coagulation cascade have shown that centhaquine does not inhibit fibrin formation, platelet accumulation, or clot lysis time [187]. Furthermore, centhaquine does not alter the anticoagulant effects of aspirin or heparin [187]. In another study, with a rabbit model of uncontrolled hemorrhagic shock, centhaquine resuscitation required less volume of fluid and resulted in greater clot strength (higher maximum amplitude values), compared to saline resuscitation [188]. These findings take on an even greater significance, because large volume resuscitation in hemorrhagic shock causes hemodilution and reduces blood viscosity and endothelial shear stress, disrupting the normal physiological mechanisms of endothelial homeostasis and promoting microcirculatory dysfunction, endothelial leak, and edema formation [189].

**Figure 5:**
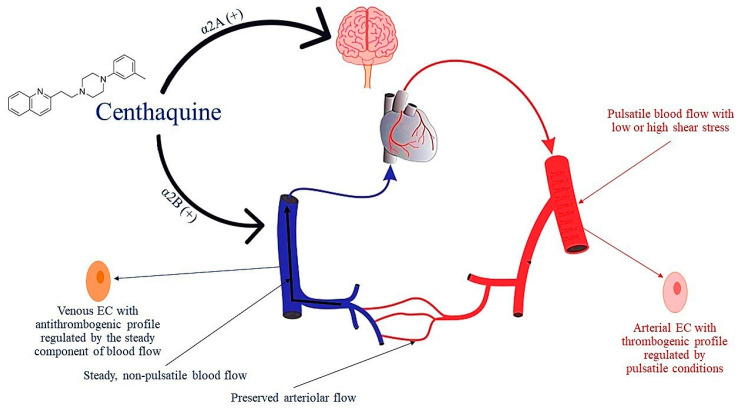
Mechanism of action of centhaquine. Centhaquine has a unique mechanism compared to other resuscitative agents. It stimulates α2B adrenergic receptors to prompt the constriction of veins, increasing the amount of blood returned to the heart. It also acts on α2A adrenergic receptors to reduce sympathetic drive, allowing arteriolar blood flow and tissue perfusion. EC, endothelial cell.

Collectively, the aforementioned basic and experimental data indicate that centhaquine does not affect the coagulation cascade and has a shear-protective action. As a pure venoconstrictor, centhaquine may maintain a steady flow and no or minimal pulsatility in veins, [30,95] exploiting the beneficial antithrombogenic profile of venous endothelial cells [184], without affecting the arterial side of circulation (Figure 5). These characteristics are important for the management of patients with hemorrhagic shock, and may prove to be of immense significance in other disease states, such as sepsis and septic shock, or in perioperative medicine.

## 8. Conclusions

The maintenance of endothelial function at the microcirculatory level is critically dependent upon hemodynamics and endothelial mechanotransduction. Normal microcirculatory flow and shear stress are necessary for homeostasis, but when they become abnormal they impair cellular/tissue physiology, causing shear stress-induced pro-coagulopathy responses and organ injury. Based on the physio- and pathophysiological mechanisms described in this article, if adequate shear stress is restored early after hemorrhage, endothelial cells could remain functional, with an intact mechanotransduction and structure (e.g., glycocalyx), maintaining an antithrombotic and anti-inflammatory phenotype. Microvascular alterations could also be treated or prevented by the development and use of innovative pharmacologic strategies that effectively target shear stress-mediated endothelial dysfunction, including shear-responsive drug delivery systems and novel antioxidants. Furthermore, targeting the venous side of the circulation using novel venoconstrictors, such as centhaquine, may facilitate the maintenance of a steady flow with no or minimal pulsatility in the veins, without affecting the arterial side of circulation, thus exploiting the beneficial antithrombogenic profile of venous endothelial cells and preventing shear-induced adverse events. Further translational research is needed to investigate the mechanisms described in the present article, leading to a more directed and balanced resuscitation and management.

## Figures and Tables

**Table 1:** Approximate normal values of diameter and shear stress in selected human blood vessels.

Vessel Type	Diameter (mm)	Shear Stress (Dynes cm^−2^)	Function
Pulmonary artery	29	5	Distribution of venous blood
Ascending aorta	25	12	Pulse dampening and distribution
Descending aorta	25	5–8	Pulse dampening and distribution
Large arteries	1.0–4.0	10–60	Distribution of arterial blood
Small arteries	0.2–1.0	10–80	Distribution and resistance
Venules	0.01–0.20	20–40	Exchange, collection, and capacitance
Veins	0.2–5.0	<5	Capacitance function (blood volume)
Vena Cava	35	<1–5	Collection of venous blood

## Data Availability

Not applicable.
